# Effect of Eyelid Opener on Wound Closure Time in Cataract Surgery

**DOI:** 10.3390/jcm14093163

**Published:** 2025-05-02

**Authors:** Takato Sakono, Naohisa Mihara, Taiji Sakamoto, Hiroto Terasaki

**Affiliations:** Department of Ophthalmology, Kagoshima University Graduate School of Medical and Dental Sciences, Kagoshima 890-8544, Japan

**Keywords:** cataract surgery, wound closure, eyelid opening, surgical efficiency, corneal endothelial health

## Abstract

**Background:** This study evaluates the effect of limiting eyelid opening on wound closure time and surgical outcomes during cataract surgery. **Methods:** This retrospective study included 129 patients who had undergone cataract surgery between December 2023 and March 2024. The patients were divided into two groups based on the degree of eyelid opening: full eyelid opening group (n = 78) and 1 mm eyelid opening group (n = 51). Wound closure time, total operation time, postoperative intraocular pressure (IOP), uncorrected and best-corrected visual acuity (UCVA and BCVA, respectively), anterior chamber protein levels, and corneal endothelial cell loss were compared between the two groups. **Results:** The 1 mm eyelid opening group had a significantly shorter wound closure time (32.9 ± 11.2 s) compared with the full eyelid opening group (57.0 ± 21.3 s, *p* < 0.001), as well as reduced total operation time (547.5 ± 83.1 s vs. 606.9 ± 144 s, *p* = 0.02). No significant differences were observed in the postoperative IOP, BCVA, UCVA, anterior chamber protein levels, or corneal endothelial cell loss between the groups. **Conclusions:** Limiting the eyelid opening to 1 mm from the corneal limbus during cataract surgery can reduce wound closure time and total operation time without compromising visual outcomes or corneal endothelial health. This simple adjustment may contribute to improved surgical efficiency.

## 1. Introduction

Cataract surgery is the most commonly performed type of ophthalmic surgery worldwide [1,2]. The widespread adoption of phacoemulsification in the 1990s marked significant advancements in surgical techniques, transitioning toward minimally invasive sutureless procedures. In recent years, phacoemulsification with corneal incisions has become standard practice [3]. Despite the improvements in surgical techniques, postoperative endophthalmitis remains one of the most severe complications of cataract surgery. The incidence of infectious endophthalmitis following cataract surgery is approximately 0.38%, with wound dehiscence observed in nearly 80% of cases. Notably, corneal incisions are associated with a three-fold higher risk of postoperative endophthalmitis than scleral incisions [4,5,6,7,8,9,10]. These findings underscore the importance of meticulous wound closure during cataract surgery.

Various techniques, including suturing, corneal welding, and stromal hydration, have been developed for this surgery [10,11,12,13,14]. However, each of these methods has inherent limitations. Suturing can lead to complications, such as high astigmatism, recurrent conjunctivitis, giant papillary conjunctivitis, and infectious endophthalmitis [15]. Corneal welding may cause visual impairment because of collagen fiber degeneration in the cornea [10]. For stromal hydration, the duration of wound closure may vary depending on the patient’s corneal endothelial function, raising concerns about the stability and reliability of this technique [10]. These limitations highlight the need for improved wound closure methods with fewer drawbacks.

While surgical factors, such as incision architecture, have been extensively studied [10,11,12,13,14], the mechanical impact of eyelid opening on wound integrity has received little attention. Excessive lid retraction may distort the corneal incision and impair its self-sealing capability. To date, no studies have systematically evaluated the relationship between the eyelid opening width and wound closure.

The degree of eyelid opening facilitated by the eyelid speculum may affect the quality of wound closure during cataract surgery. Therefore, in this study, the eyelid opening width was measured intraoperatively and the postoperative condition of the incision was observed to assess the relationship between the eyelid opening and wound stability.

## 2. Material and Methods

### 2.1. Ethics Statement

All procedures used in this study conformed to the tenets of the Declaration of Helsinki and were approved by the Ethics Committee of Kagoshima University Hospital. The requirement for patient consent was waived owing to the retrospective nature of the study and the use of anonymized clinical data. The research plan has been published on the homepage of our hospital’s website and guaranteed an opt-out opportunity according to the instructions of the committee.

### 2.2. Subjects

This retrospective study was conducted at our institution and included 129 patients who underwent cataract surgery between December 2023 and March 2024. All surgeries were performed by a cataract specialist (TS) with extensive experience, and over 2000 cataract surgeries were performed.

### 2.3. Surgical Methods

**Figure 1:**
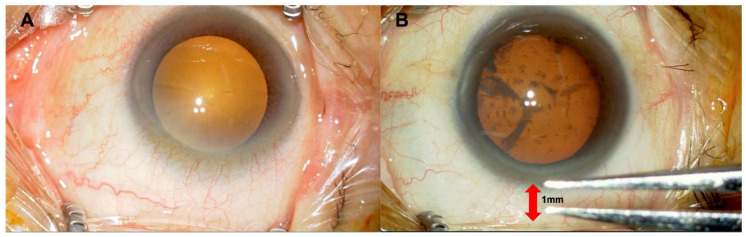
Surgeon’s view. (**A**). Maximum eyelid retraction with an eyelid speculum. (**B**). Eyelid speculum positioned 1 mm from the corneal limbus.

Patients were categorized into two groups based on the degree of eyelid opening during surgery: (i) full eyelid opening group (n = 78): Cataract surgery was performed using the Bungter eyelid retractor (Inami, Japan) adjusted to achieve maximum eyelid opening for each patient (Figure 1A); (ii) 1 mm eyelid opening group (n = 51): Cataract surgery was performed with the eyelid retractor adjusted to maintain a 1 mm gap from the corneal limbus (Figure 1B).

To define the 1 mm eyelid opening condition, the distance from the tip of the eyelid speculum to the limbus was measured using a Gastroviejo Caliper (INAMI, Tokyo, Japan) at the beginning of surgery.

The CENTURION Vision System with an ACTIVE SENTRY (Alcon Japan Ltd., Tokyo, Japan) was used for all procedures. Ophthalmic viscosurgical devices (OVD) included Shelgan 0.5, ophthalmic viscoelastic material and sodium hyaluronate 1% ophthalmic viscoelastic material (Santen, Tokyo, Japan). The intraocular lenses (IOLs) used were the XY-1 and XY-1 Toric (Vivinex, HOYA, Tokyo, Japan). The total operation time was defined as the duration from the initiation of the main surgical incision to the completion of wound closure. Wound closure time was defined as the period from the removal of the OVD to the completion of wound sealing.

During this interval, only intraocular pressure (IOP) adjustment was performed, and no additional intraoperative procedures were required in any of the cases. Incisions were made using a 2.2-mm slit knife (Takumi slit knife, KAI MEDICAL, Toyo, Japan) via a single-plane transconjunctival approach.

### 2.4. Comparison Between Groups: Full Eyelid Opening Group and 1-mm Eyelid Opening Group

All patients underwent comprehensive ophthalmic examinations, including slit-lamp biomicroscopy and fundus evaluation, and were deemed suitable candidates for cataract surgery. Preoperative assessments included best-corrected visual acuity (BCVA) measured at 5 m using a Landolt C chart displayed on a digital visual acuity system (System Chart SC-1600; NIDEK, Tokyo, Japan), intraocular pressure (IOP) measured with a non-contact tonometer (NCT; NT-1, NIDEK, Japan), corneal endothelial cell density measured using a specular microscope (CEM-530 PARACENTRAL; NIDEK, Japan), axial length measured with an optical biometer (IOLMaster; Carl Zeiss Meditec, Jena, Germany), and objective refraction and keratometry assessed using an autorefractor-keratometer (KR-1W; Topcon, Tokyo, Japan).

During cataract surgery, the time from the removal of the OVD from the anterior chamber to the completion of surgery was recorded as the wound closure time, following the removal of the crystalline lens nucleus and cortical material, and the insertion of the IOL. The postoperative IOP was evaluated on days 1 and 2 using a non-contact tonometer. Additionally, on postoperative days 2 and 7, uncorrected and best-corrected visual acuity were assessed, and anterior chamber protein levels were measured using an anterior chamber protein measurement device (FM600α, Kowa, Tokyo, Japan). These parameters were then compared between the two groups.

On postoperative day 7, the corneal endothelial cell density was measured using CEM-530 PARACENTRAL (NIDEK, Japan). The reduction in corneal endothelial cell density from the preoperative levels was compared between the two groups to evaluate the impact of the degree of eyelid opening during surgery on endothelial health and surgical outcomes.

### 2.5. Statistical Analysis

All continuous variables measured pre- and postoperatively were summarized as mean ± standard deviation (SD). Normality of the data distribution was assessed using the Shapiro–Wilk test. Since normality could not be confirmed for most variables and the small sample size limited the applicability of parametric tests, comparisons between the 1 mm eyelid opening group and the maximum eyelid opening group were performed using the non-parametric Mann–Whitney U test. Categorical variables were compared using the chi-squared test. A *p*-value of less than 0.05 was considered statistically significant. Where applicable, 95% confidence intervals (CIs) were calculated and reported to enhance the interpretability of the findings. For each patient, one eye was randomly selected for inclusion in the analysis to avoid inter-eye correlation. Statistical analyses were performed using SPSS for Windows (SPSS Inc., IBM, Somers, New York, NY, USA) and EZR (Saitama Medical Center, Jichi Medical University, Saitama, Japan), which is a graphical user interface for R [16].

## 3. Results

### 3.1. Subjects Characteristics

The preoperative patient demographic data are summarized in Table 1. In the full eyelid opening group, there were 78 eyes (43 male and 35 female, 41 right eyes and 37 left eyes), with a mean age of 71.6 ± 9.40 years. The mean BCVA was −0.21 ± 0.37 logMAR, and the mean refractive error was −1.90 ± 4.59 diopters. The mean axial length was 24.1 ± 3.09 mm, and the mean corneal endothelial cell count was 2744 ± 263 cells/mm^2^.

In the 1 mm eyelid opening group, there were 51 eyes (21 male and 30 female, 24 right eyes and 27 left eyes), with a mean age of 72.9 ± 7.30 years. The mean BCVA was −0.16 ± 0.43 logMAR, and the mean refractive error was −0.38 ± 2.95 diopters. The mean axial length was 24.2 ± 1.12 mm, and the mean corneal endothelial cell count was 2746 ± 251 cells/mm^2^.

There were no significant intergroup differences in age, preoperative BCVA, axial length, or corneal endothelial cell count between the two groups.

### 3.2. Comparison of Postoperative Ocular Data

The wound closure times and postoperative data for the two groups are summarized in Table 2. In the full eyelid opening group, the postoperative day 2 BCVA was −0.09 ± 0.18 logMAR, and the postoperative day 7 BCVA was −0.016 ± 0.12 logMAR. The IOP on postoperative day 1 was 15.7 ± 4.50 mmHg, and on day 2 it was 13.7 ± 3.41 mmHg. The anterior chamber protein levels were 17.7 ± 9.75 on postoperative days 2 and 13.3 ± 6.47 on day 7. The corneal endothelial cell density on postoperative day 7 was 2770 ± 301 cells/mm^2^, and the reduction in corneal endothelial cell density was −25.5 ± 279 cells/mm^2^, the total operation time was 606.9 ± 144 s, and the wound closure time was 57.0 ± 21.3 s.

In the 1 mm eyelid opening group, the postoperative day 2 BCVA was −0.08 ± 0.15 logMAR, and the postoperative day 7 BCVA was −0.002 ± 0.10 logMAR. The IOP on postoperative day 1 was 15.1 ± 3.28 mmHg, and on day 2 it was 13.8 ± 2.81 mmHg. The anterior chamber protein levels were 17.6 ± 6.82 on postoperative day 2 and 12.8 ± 5.95 on day 7. The corneal endothelial cell density on postoperative day 7 was 2686 ± 284 cells/mm^2^, and the reduction in corneal endothelial cell density was 53.9 ± 168 cells/mm^2^, the total operation time was 547.5 ± 83.1 s, and the wound closure time was 32.9 ± 11.2 s.

Improvement in uncorrected visual acuity (UCVA) is closely linked to patient satisfaction after cataract surgery. In this study, postoperative anterior chamber protein levels were compared between days 2 and 7 to assess postoperative inflammation. However, no significant differences were observed between the two groups on day 2 or 7 (*p* = 0.904 and 0.354, respectively; Table 2).

Additionally, a comparison was made of the UCVA on postoperative days 2 and 7 in patients who desired a postoperative refractive error of ±0D. No significant differences were observed between the two groups, indicating that the improvements in UCVA were similar. Furthermore, there was no evidence of inferior UCVA recovery rates between the groups (*p* = 0.881 and 0.910, respectively; Table 2).

The total operation time (*p* = 0.02) and wound closure time (*p* < 0.001) were significantly shorter in the 1 mm eyelid opening group than in the maximum eyelid opening group. There were no significant differences between the groups in terms of BCVA, IOP, anterior chamber protein levels, corneal endothelial cell density, or reduction from preoperative values.

### 3.3. Comparison of Wound Closure and Total Operation Time

**Figure 2:**
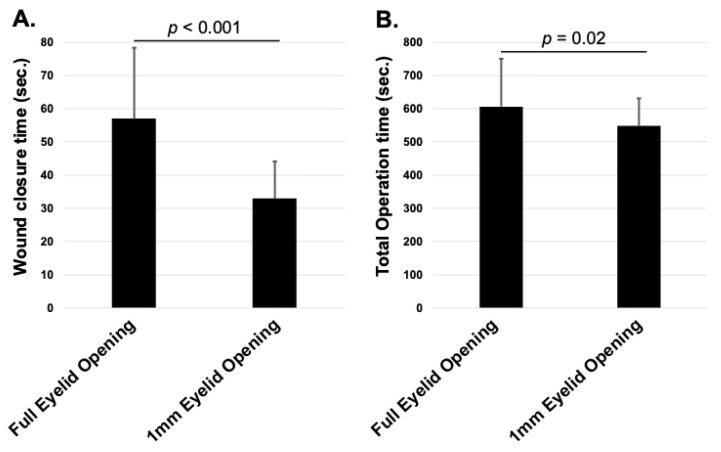
Comparison of wound closure time and total operation time in cataract surgery between the full eyelid opening group and the 1 mm eyelid opening group. (**A**) Wound closure time: The 1 mm group showed significantly shorter closure time (*p* < 0.001). (**B**) Total operation time: The 1 mm group also had significantly shorter total time (*p* = 0.02).

The wound closure time (*p* < 0.001, Figure 2A) and total operation time (*p* = 0.02, Figure 2B) were significantly shorter in the 1 mm eyelid opening group than in the maximum eyelid opening group.

## 4. Discussion

The study demonstrated that the wound closure time was significantly shorter in the 1 mm eyelid opening group than in the maximum eyelid opening group, suggesting that limiting eyelid opening with a retractor may facilitate the closure of scleral incisions.

Uncorrected visual acuity (UCVA) is strongly associated with corneal astigmatism. Approximately 75–85% of individuals are reported to have some degree of corneal astigmatism, with 20–25% having corneal astigmatism of 1.5 diopters or greater [17,18]. Corneal astigmatism tends to follow the with-the-rule (WTR) in younger individuals and shifts to the against-the-rule (ATR) with aging [10]. Consequently, superior incisions in cataract surgery in elderly patients may have a negative impact on UCVA because of surgically induced astigmatism. Smaller incisions are recommended to minimize this effect [19,20,21,22,23]. While 2.2 mm incisions are technically more challenging to create as square-shaped wounds than larger 2.65-mm incisions, they induce less astigmatism. However, some studies report that 2.2 mm incisions may be inferior in terms of wound closure [11,19,20,21,22,23]. The findings of this study suggest that this simple and practical technique may enhance the effectiveness of 2.2 mm micro-incision cataract surgery by providing a safe approach that reduces surgically induced astigmatism and improves postoperative UCVA.

**Figure 3:**
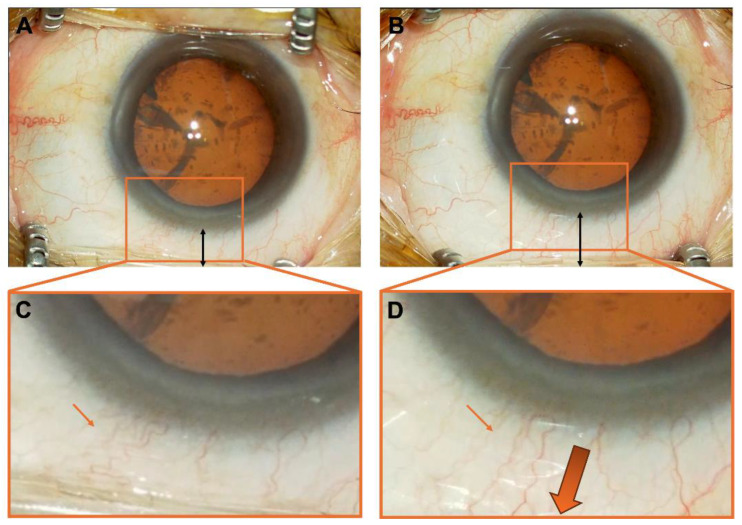
Changes in conjunctival vessels depending on the condition of the eyelid retractor. (**A**). State with the eyelid speculum retracted 1 mm from the limbus. (**B**). State with the eyelid speculum fully retracted to its maximum extent. (**C**). Enlarged view of (**A**) Thin arrows indicate minimally stretched and tortuous conjunctival vessels. (**D**). Enlarged view of (**B**) Thin arrows indicate stretched and elongated conjunctival vessels under tension, while thick arrows represent the direction of conjunctival traction.

This study found no significant differences in preoperative background factors between the group that underwent cataract surgery with a 1 mm eyelid opening and the group with a maximum eyelid opening. This indicates that the traction caused by excessive eyelid opening may influence wound closure. Observation of the conjunctival vessels under varying degrees of eyelid opening revealed that a weaker eyelid opening resulted in relaxed and tortuous conjunctival vessels, whereas a stronger eyelid opening caused the vessels to stretch (Figure 3). This suggests that excessive eyelid opening may create traction opposing wound closure, whereas reduced eyelid opening may facilitate favorable conditions for wound closure.

### 4.1. Advantages

The proposed technique offers several advantages. Unlike sutured wound closure, which can lead to complications, such as high astigmatism and infections [10,13], this approach eliminates these risks. Wound closure using stromal hydration may cause visual impairment due to increased corneal edema or anterior chamber reactions and may also increase the risk of Descemet’s membrane detachment [10]. In this study, none of the patients required suturing and the use of stromal hydration for wound closure was minimized.

Furthermore, this technique does not require additional procedures or devices other than adjusting the eyelid retractor width, contributing to a shorter wound closure and total operation time. These results underscore the potential utility of limiting eyelid opening to achieve more effective wound closure and improve overall surgical efficiency without additional complications.

However, some challenges remain unresolved. During cataract surgeries performed with a 1 mm eyelid opening from the corneal limbus, there were a few rare instances in which surgical instruments, such as an ultrasonic (US) handpiece, interfered with the eyelid retractor or where the retractor itself became displaced. Although these occurrences did not affect the surgery or its outcomes in this study, they may pose a potential risk for surgical complications, particularly for less-experienced cataract surgeons.

Furthermore, if an eyelid retractor applies vertical traction to the eyeball, the retractor itself could potentially aid wound closure in cases involving temporal corneal incision techniques. However, further investigation is required to better understand its implications and benefits in surgical practice.

### 4.2. Limitations

This study has several limitations inherent to its retrospective design. First, the sample size was relatively small, and the follow-up period was short, limiting long-term comparisons. In addition, only two eyelid opening widths (1.0 mm and the maximum observed intraoperatively) were available for analysis, and a more detailed prospective evaluation across multiple controlled opening widths is warranted in future studies. All procedures were performed by experienced surgeons, and no significant displacement of the eyelid speculum was observed; however, minor intraoperative shifts could have occurred and may have been more influential in less experienced hands. Furthermore, the sample size imbalance between the groups, which reflects the timing when the 1 mm eyelid opening approach was incorporated into clinical practice, and the use of a single incision width (2.2 mm) may have affected the wound closure outcomes. Notably, given that a difference was observed even with a 2.2 mm incision, larger incisions (e.g., 2.4 or 2.7 mm) may further compromise wound stability under a wide eyelid opening. Finally, potential confounding factors such as ocular surface status, incision architecture, and intraoperative hydration technique could not be fully controlled. In addition, no formal adjustment for multiple comparisons was applied because of the exploratory nature and small sample size of this study.

## 5. Conclusions

Performing cataract surgery with eyelid opening limited to 1 mm from the corneal limbus was associated with a shorter wound closure time in this study. Although the clinical relevance of this time difference requires further investigation, this approach may contribute to improved surgical efficiency without increasing complications.

## Figures and Tables

**Table 1:** Preoperative patient demographics.

	Full Eyelid Opening Group (Mean ± SD)	1 mm Eyelid Opening Group (Mean ± SD)	*p* Value
Age	71.6 ± 9.40	72.9 ± 7.30	0.904
Right/left	41/37	24/27	0.666
Sex (male/female)	43/35	21/30	0.171
BCVA (logMAR)	−0.21 ± 0.37	−0.16 ± 0.43	0.354
Refractive error (diopter)	−1.90 ± 4.59	−0.38 ± 2.95	0.266
Axial length (mm)	24.1 ± 3.09	24.2 ± 1.12	0.925
Corneal endothelium (cells/mm^2^)	2744 ± 263	2746 ± 251	0.747

SD: standard deviation, BCVA: best corrected visual acuity.

**Table 2:** Comparison of postoperative data and wound closure time between groups.

	Full Eyelid Opening Group (Mean ± SD)	95% CI	1 mm Eyelid Opening Group (Mean ± SD)	95% CI	*p* Value
Postoperative day 2 BCVA (logMAR)	−0.09 ± 0.18	−0.13–−0.049	−0.08 ± 0.15	−0.12–0.032	0.588
Postoperative day 7 BCVA (logMAR)	−0.016 ± 0.12	−0.044–0.012	−0.002 ± 0.10	−0.030–0.031	0.231
Postoperative day 2 UCVA (logMAR)	−0.19 ± 0.15	−0.24–−0.15	−0.20 ± 0.24	−0.28–0.12	0.881
Postoperative day 7 UCVA (logMAR)	−0.11 ± 0.16	−0.15–−0.066	−0.10 ± 0.20	−0.16–0.032	0.910
Intraocular pressure on postoperative day 1 (mmHg)	15.7 ± 4.50	14.7–16.7	15.1 ± 3.28	14.0–15.9	0.652
Intraocular pressure on postoperative day 2 (mmHg)	13.7 ± 3.41	13.0–14.5	13.8 ± 2.81	12.9–14.4	0.915
Anterior chamber protein measurement day 2	17.7 ± 9.75	15.4–20.0	17.6 ± 6.82	15.3–19.3	0.904
Anterior chamber protein measurement day 7	13.3 ± 6.49	11.7–14.8	12.8 ± 5.95	10.9–14.6	0.354
Postoperative day 7 corneal endothelium (cells/mm^2^)	2770 ± 301	2700–2838	2686 ± 284	2605–2767	0.095
Postoperative corneal endothelial reduction (cells/mm^2^)	−25.5 ± 279	−89.3–38.3	53.9 ± 168	−0.29–121	0.158
Total operation time (second)	606.9 ± 144	574–640	547.5 ± 83.1	523–571	0.02
Wound closing time (second)	57.0 ± 21.3	52.1–61.9	32.9 ± 11.2	31.7–38.2	<0.001

SD, standard deviation; CI, confidence interval; BCVA, best-corrected visual acuity; UCVA, uncorrected visual acuity.

## Data Availability

The data presented in this study are available on request from the corresponding author. The data are not publicly available due to privacy and ethical restrictions.
